# Extracellular heat shock protein 90α mediates HDM-induced bronchial epithelial barrier dysfunction by activating RhoA/MLC signaling

**DOI:** 10.1186/s12931-017-0593-y

**Published:** 2017-05-30

**Authors:** Hang-ming Dong, Yan-qing Le, Yan-hong Wang, Hai-jin Zhao, Chao-wen Huang, Ya-hui Hu, Li-shan Luo, Xuan Wan, Yi-lan Wei, Zi-qiang Chu, Wei Li, Shao-xi Cai

**Affiliations:** 1 Chronic Airways Diseases Laboratory, Department of Respiratory and Critical Care Medicine, NanFang Hospital, Southern Medical University, Guangzhou, 510515 China; 2 Department of Dermatology and the Norris Comprehensive Cancer Centre, University of Southern California Keck, Medical Centre, Los Angeles, CA 90033 USA

**Keywords:** Bronchial epithelial, Barrier dysfunction, Extracellular heat shock protein90α, RhoA/MLC signaling, House dust mite

## Abstract

**Background:**

The disruption and hyperpermeability of bronchial epithelial barrier are closely related to the pathogenesis of asthma. House dust mite (HDM), one of the most important allergens, could increase the airway epithelial permeability. Heat shock protein (Hsp) 90α is also implicated in the lung endothelial barrier dysfunction by disrupting RhoA signaling. However, the effect of extracellular Hsp90α (eHsp90α) on the bronchial epithelial barrier disruption induced by HDM has never been reported.

**Methods:**

To investigate the involvement of eHsp90α in the bronchial epithelial barrier disruption induced by HDM, normal human bronchial epithelial cell line 16HBE14o- (16HBE) cells were treated by HDM, human recombinant (hr) Hsp90α and hrHsp90β respectively and pretreated by1G6-D7, a specific anti-secreted Hsp90α monoclonal antibody (mAb). Hsp90α-silencing cells were also constructed. To further evaluate the role of RhoA signaling in this process, cells were pretreated by inhibitors of Rho kinase, GSK429286A and Y27632 2HCl. Transepithelial electrical resistance (TEER) and FITC-dextran flux (FITC-DX) were examined as the epithelial barrier function. Expression and localization of adherens junctional proteins E-cadherin and β-catenin were evaluated by western blotting and immunofluorescence respectively. The level of eHsp90α was investigated by concentration and purification of condition media. RhoA activity was determined by using a Rho G-LISA® RhoA activation assay kit^TM^ biochem kit, and the phosphorylation of myosin light chain (MLC), the downstream signal molecule of RhoA, was assessed by western blotting.

**Results:**

The epithelial barrier disruption and the loss of adherens junctional proteins E-cadherin and β-catenin in cytomembrane were observed in HDM-treated 16HBE cells, paralleled with the increase of eHsp90α secretion. All of which were rescued in Hsp90α-silencing cells or by pretreating 16HBE cells with 1G6-D7. Also, 1G6-D7 suppressed RhoA activity and MLC phosphorylation induced by HDM. Furthermore, inhibitors of Rho kinase prevented and restored the airway barrier disruption. Consistently, it was hrHsp90α instead of hrHsp90β that promoted barrier dysfunction and activated RhoA/MLC signaling in 16HBE cells.

**Conclusions:**

The eHsp90α mediates HDM-induced human bronchial epithelial barrier dysfunction by activating RhoA/MLC signaling, suggesting that eHsp90α is a potential therapeutic target for treatment of asthma.

## Background

The airway epithelium forms a continuous, highly regulated physical barrier lining of the airway lumen, prevents invasion of inhaled environmental agents such as aeroallergens, pollutants and pathogens [1]. Intercellular junctions are comprised of tight junctions (TJs), adherens junctions (AJs) and desmosomes [1]. Presence of dysfunctional epithelial barrier in the asthmatic airway included hyperpermeability to allergens and disrupted expression of the junction molecule E-cadherin at sites of epithelial detachment [2, 3]. The decrease of epithelial E-cadherin protein expression in patients with atopic asthma contributed to defective airway epithelial barrier [4, 5]. E-cadherin/β-catenin complex, the AJs complex (AJC), plays an important role in maintaining epithelial integrity and disrupting this complex affect not only the adhesive repertoire of a cell, but also the Wnt-signaling pathway [6], leading to the initiation and chronicity of asthma. Furthermore, dysregulation of epithelial repair in asthmatic airways, including epithelial-mesenchymal transition, contributed to asthmatic airway remodeling [7].

Heat shock protein (Hsp) 90 is an essential molecular chaperone in eukaryotic cells with an important role in activation and maintenance of numerous regulatory and signaling proteins [8]. Since many of Hsp90 clients are oncogenic proteins, Hsp90 has become a therapeutic target for treatment of cancer. However, tumor cells have managed to constitutively secrete Hsp90 for tissue invasion, whereas normal cells also secrete Hsp90 in response to tissue injury [9]. Abundant evidences also showed that Hsp90 inhibitors protected and restored pulmonary endothelial barrier dysfunction [10–12]. Furthermore, Tong W, et al. reported that higher expression of Hsp90 mRNA and protein were detected in asthmatic patients than healthy controls [13, 14]. Hsp90 contributed to deterioration of airway inflammation in an ovalbumin-induced murine asthma model [15]. Also, inhibition of Hsp90 decreased the relaxation of tracheal ring in asthmatic mice [16]. Fundamental distinctions between intracellular and extracellular Hsp90 are clear. Both Hsp90α isoform and Hsp90β isoform can be secreted to extracellular space. Hsp90β mainly acts as a molecular chaperone protein that maintains the intracellular signaling networks essential for life [17]. Mice lacking Hsp90β failed to develop a placental labyrinth [18]. However, Hsp90α exerts pro-mobility signal after being secreted to extracellular environment by cells stimulated with heat [19], hypoxia [20, 21], gamma-irradiation [21] or tissue injury-released cytokines, such as TGF-α [22]. Extracellular Hsp90 participated in TGF-β-mediated collagen production in myocardial fibroblasts [23], wound healing in keratinocytes [9, 24] and contributed to tumor growth, invasion, and inflammatory storm [9, 25–29]. These findings prompted us to investigate the role of extracellular Hsp90α (eHsp90α) in HDM-induced airway barrier dysfunction. A newly generated mAb, 1G6-D7, which selectively targets the dual lysine region in secreted Hsp90α, represents a viable anticancer agent [30, 31]. Therefore, we also used 1G6-D7 in this study to evaluate the role of eHsp90α in airway barrier disruption.

The Rho family of small GTPases plays an important role in a variety of cellular responses, including contraction, motility and proliferation and barrier dysfunction [32–34]. Rho kinase (ROCK) is activated by RhoA-GTP, and induces phosphorylation of myosin light chain (MLC), the downstream molecule of Rho. Then phosphorylated MLC regulates cellular contraction and promotes cytoskeletal reorganization [11, 35, 36]. Studies revealed that RhoA signaling was involved in intestinal and prostate epithelial barrier hyperpermeability [37, 38]. Hyaluronan and layilin mediated loss of airway epithelial barrier function induced by cigarette smoke by Rho/ROCK signaling to decrease E-cadherin, leading to a loss of epithelial cell-cell contact [39]. Moreover, inhibition of Hsp90 prevented LPS-induced lung endothelial barrier dysfunction by disrupting RhoA signaling [11], suggesting a role for Hsp90 in regulating RhoA signaling. These indicated that activated RhoA signaling resulted in increased epithelial and endothelial permeability. Thus, we hypothesized that eHsp90α mediated HDM-induced bronchial epithelial barrier dysfunction via activating RhoA/MLC signaling.

## Methods

### Antibodies and reagents

HDM, 100000 SQ-U (U)/ml in Alutard, was purchased from ALK-Abello A/S (Guangzhou, China). 1G6-D7 (noncommercial antibody), the anti-secreted Hsp90α mAb, was kindly granted by the University of Southern California Keck School of Medicine in USA. The Rho kinase inhibitors, Y-27632 2HCl (S1049) and GSK429286A (S1474), were obtained from Selleck Chemicals (Shanghai, China). Human recombinant (hr) Hsp90α (SPR-101C) and hrHsp90β (SPR-102C) were purchased from Stressmarq Bioscience (Victori, Canada). Anti-β-actin(8457), anti-MLC(3672), anti-phospho(p)-MLC(3674) antibodies and horseradish peroxidase (HRP)-conjugated donkey anti-rabbit or anti-mouse IgG antibodies were purchased from Cell Signaling Technology (Beverly, USA). Anti-Hsp90αantibody (CA1023) was obtained from Calbiochem (Billerica, USA) and anti-Hsp90β antibody (SMC 107) was from Stressmarq Biosciences (Victoria, Canada). Anti-E-cadherin (sc-7870) and anti-β-catenin (sc-7199) antibodies were purchased from Santa Cruz Biotechnologies (Santa Cruz, USA). Alexa 488-labeled goat anti-rabbit secondary antibody was from Life Technologies (Grand Island, USA).

### Cell culture and treatment

Human bronchial epithelial cell line 16HBE14o- (16HBE) cells were purchased from Bio-Rad Biological Technology Co. Ltd (Shanghai, China), which was the agent of ATCC. Cells were cultured in RPMI-1640 medium containing 10%fetal calf serum at 37 °C and 5% CO_2_ incubator. When reaching 90% confluence, the cells were seeded to proper culture plates at a density of 10^4^–10^5^ cells/cm^2^. After 24 h incubation, the culture medium was changed into serum free RPMI-1640. Different concentrations of HDM, hrHsp90α or hrHsp90β were added to the culture medium for indicated times partly based on previous studies [17, 40, 41].

### Measurement of transepithelial electrical resistance (TEER) and FITC-dextran flux (FITC-DX)

Confluent monolayers of 16HBE cells were cultured in 12-well Transwell inserts, purchased from Corning Company. Based on previous studies [42, 43], cells were seeded into 12-well transwell inserts duplicates (density of 2*10^5^/well) and complete confluence was reached almost for 3 additional days. TEER was measured by using a Millicell ERS-2 Epithelial Volt-Ohm meter with an STX01 electrode (MA, USA), and the levels were normalized to medium only (con) group.

Confluent monolayers of 16HBE cells were cultured in 24-well Transwell inserts. Cells were seeded into 24-well transwell inserts duplicates (density of 1 *10^5^/well) and complete confluence was reached almost for 3 additional days. FITC-DX, purchased from Sigma Company, was added to the upper chamber followed by incubation for 1 h at 37 °C before the 100 μl liquid from both chambers was removed. Fluorescence values were measured by using a fluorescent plate reader (Männedorf, Switzerland). The values of FITC-DX permeability were normalized to the con group, which was denoted by Pa/Pc%.

### Immunofluorescence staining

The cell monolayers were grown on glass bottom cell culture dishes (Guangzhou, China) and were fixed in 4% paraformaldehyde at room temperature for 30 min before washing with PBS (5 min × 3 times), then incubated with 0.2% Triton X-100 in PBS for 10 min, and washed again with PBS. The cells were blocked with 5% skim milk in PBS for 3 h and then incubated overnight with a primary antibody (E-cadherin or β-catenin) at 4 °C. Then cell monolayers were incubated with alexa 488-labeled goat anti-rabbit secondary antibody for 1 h at 37 °C in the dark and washed again with PBS. 4′, 6-diamidino-2-phenylindole dihydrochloride (DAPI) (Shanghai, China) was added to stain the cell nucleus for 10 min. A laser scanning confocal microscope (Tokyo, Japan) was used to examine the location of E-cadherin and β-catenin in the 16HBE cells.

### RhoA activity assay

RhoA activity was determined by using a Rho G-LISA® RhoA activation assay kit^TM^ biochem kit according to the manufacturer’s instructions (Denver, USA). Results were normalized to protein levels measured by the Precision Red protein assay reagent.

### Concentration and purification of condition media

The condition media of 16HBE cells treated by HDM was collected for centrifugation at 1500 *g* for 5 min. Then the supernatants were added to centrifugal filter units from Millipore Company (Bedford, MA) for concentration and purification following the manufacturer’s instructions. Finally the concentrated and purified supernatants were prepared for detecting the level of eHsp90α protein by Western blotting analysis.

### Lentiviral systems for silencing of Hsp90α protein

Lentiviral systems for knocking down the Hsp90α gene were completed by Hanbio Biotechnology Co. Ltd (Shanghai, China). The cDNAs sequence for human Hsp90α was 5′-GGAAAGAGCTGCATATTAA-3′. Wild-type or mutant Hsp90α cDNAs was inserted into the lentivirus-derived vector. These constructs were used to transfect 16HBE cells. The transfect 16HBE cells were respectively named Sh-Hsp90α and Sh-LacZ cells. Silencing of the endogenous Hsp90α protein product was identified by western blotting analysis.

### Western blotting analysis

To evaluate the protein expression of Hsp90α, Hsp90β, E-cadherin, β-catenin, MLC, and p-MLC, Total cell lysates were subjected to 10% or 12% SDS-PAGE, transferred to PVDF membrane (Bedford, MA), and then probed with the following antibodies: Hsp90α, Hsp90β, MLC, p-MLC, E-cadherin and β-catenin. After incubation with an IRDye®680WC-conjugated secondary antibody (LI-COR Biosciences), immunoreactive bands were exposed to Odyssey® CLx Imager for image capture. Or after incubation with horseradish peroxidase (HRP)-conjugated donkey anti-rabbit or anti-mouse IgG antibody, the immunoreactive bands were detected using the enhanced chemiluminescence from Millipore Company (Bedford, MA). Quantitative image analysis was performed with Image J software.

### Statistical analysis

SPSS 20.0 software was used for the statistical analyses. Statistical analysis was performed by one-way analysis of variance (ANOVA) and post hoc tests were analyzed by Bonferonni (equal variances assumed) or Dunnett’s T3 (equal variances not assumed) post hoc tests for multiple comparisons. Results were presented as mean ± standard deviations (SD). *P* <0.05 was considered to be statistically significant. At least three independent experiments were repeated.

## Results

### HDM induced bronchial epithelial hyperpermeability

**Fig. 1:**
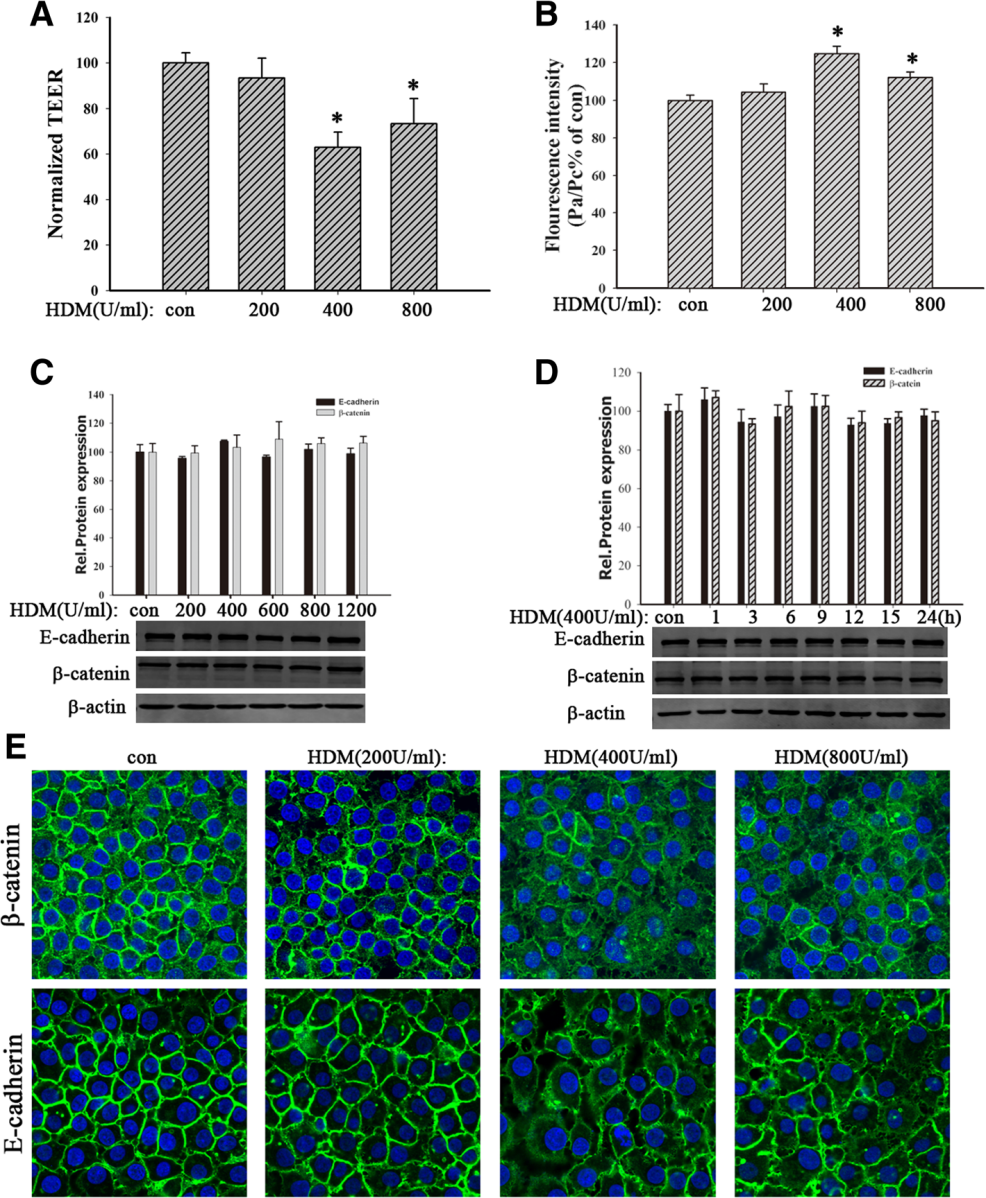
HDM induced bronchial epithelial hyperpermeability and E-cadherin and β-catenin delocalization. **a** and **b**) 16HBE cells were exposed to HDM (200, 400, or 800 U/ml) for 24 h. TEER values and FITC-DX were measured immediately. **c** and **d**) 16HBE cells were treated with HDM (200, 400, 600, 800 or 1200 U/ml) for 24 h or stimulated with HDM (400 U/ml) for 1, 3, 6, 9, 12, 15 or 24 h. The expression of E-cadherin and β-catenin proteins was detected by Western blotting analysis in total lysates. **e** The location of E-cadherin and β-catenin was detected by immunofluorescence staining. Data are mean ± SD of four independent experiments. ^*^ *P* < 0.05 versus con group

To clarify the effect of HDM on loss of bronchial epithelial barrier integrity, 16HBE cells were stimulated with different concentrations of HDM (200, 400, 800 U/ml) for 24 h, which were used for measurement of TEER and FITC-DX. Results indicated that HDM (400 U/ml or 800 U/ml) induced the fall of TEER values (Fig. 1a, *P* < 0.05) and the raise of FITC-DX (Fig. 1b, *P* < 0.05) compared to con group, suggesting that HDM induced bronchial epithelial hyperpermeability.

### HDM induced delocalization of E-cadherin and β-catenin

E-cadherin/β-catenin complex plays an important role in maintaining epithelial integrity [6]. Here we further detected the effect of HDM on E-cadherin/β-catenin complex in 16HBE cells. Cells were exposed to different concentrations of HDM (200, 400, 800 U/ml) for 24 h. Western blotting analysis revealed that HDM treatment did not affect the expression of E-cadherin or β-catenin in 16HBE cells (Fig. 1c-d, *P* > 0.05). However, the immunofluorescence showed that HDM promoted delocalization of E-cadherin and β-catenin, exhibiting discontinuous and diffusing from the adjacent cell borders to cytoplasm (Fig. 1e). These results indicated that HDM induced the abnormal distribution of E-cadherin and β-catenin in 16HBE cells. It suggested that HDM could induce bronchial epithelial barrier dysfunction by disrupting the localization of E-cadherin/β-catenin complex in cell-cell contact.

### HDM promoted the expression and secretion of Hsp90α in 16HBE cells

**Fig. 2:**
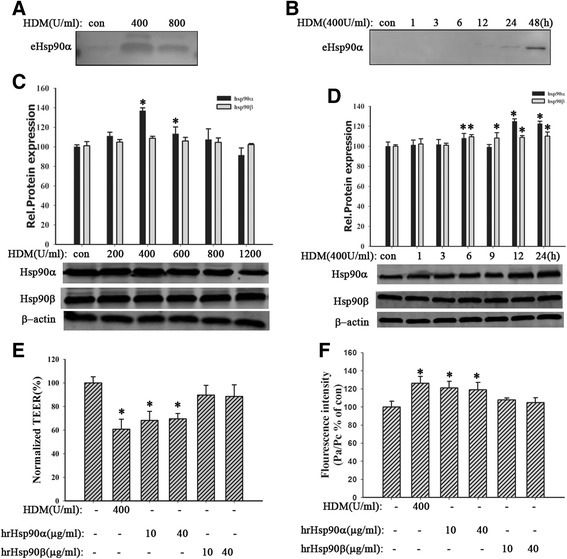
HDM promoted the expression and secretion of Hsp90α and hrHsp90α induced bronchial epithelial hyperpermeability. **a** and **b** 16HBE cells were exposed to HDM (400 or 800 U/ml) for 24 h or stimulated with HDM (400 U/ml) for 1, 3, 6, 12, 24, or 48 h. The supernatants were collected for detecting eHsp90α by western blotting analysis. **c** and **d** 16HBE cells were treated with HDM (200, 400, 600, 800, or 1200 U/ml) for 24 h or stimulated with HDM (400 U/ml) for 1, 3, 6, 9, 12, or 24 h. The expression of Hsp90α and Hsp90β was detected by western blotting analysis. **e** and **f** Cells were exposed to HDM (400 U/ml), hrHsp90α (10, 40 μg/ml), or hrHsp90β (10, 40 μg/ml) for 24 h. TEER values and FITC-DX were measured. Data are mean ± SD of three independent experiments.^*^ *P* < 0.05 versus con group

Normal cells could secrete Hsp90α in response to injury stimulations [9]. Thus, we investigated whether HDM induced secretion of Hsp90α in 16HBE cells. Cells were stimulated by different concentrations of HDM (200, 400, 800 U/ml) for 24 h or by HDM (400 U/ml) for indicated time (1, 3, 6, 12, 24 or 48 h). Then the supernatants were collected for concentration and purification to detect eHsp90α. Western blotting analysis revealed that HDM induced a significant increase of Hsp90α at 400 U/ml (Fig. 2a). The eHsp90α protein was detected from 12 h and accumulated in a time-dependent manner (Fig. 2b). It suggested that HDM promoted the secretion of Hsp90α. Besides, we extracted the total cell lysates to detect the level of intracellular Hsp90α. Results showed the intracellular Hsp90α also was increased in HDM-exposed cells (Fig. 2c-d, *P* < 0.05). Therefore, these data indicated that HDM increased both intracellular and extracellular Hsp90α protein expression in 16HBE cells.

### HrHsp90α induced bronchial epithelial hyperpermeability

To identify whether Hsp90α contributed to HDM-induced airway epithelial barrier disruption, cells were treated by hrHsp90α (10, 40 μg/ml) or hrHsp90β (10, 40 μg/ml) for 24 h. Results indicated that it was hrHsp90α instead of hrHsp90β that induced the fall of TEER values (Fig. 2e, *P* < 0.05) and the raise of FITC-DX (Fig. 2f, *P* < 0.05), suggesting hrHsp90α induced bronchial epithelial hyperpermeability. Collectively, these data indicated that Hsp90α probably played a critical role in HDM-induced bronchial epithelial barrier dysfunction.

### Hsp90α-silencing alleviated HDM-induced barrier dysfunction

**Fig. 3:**
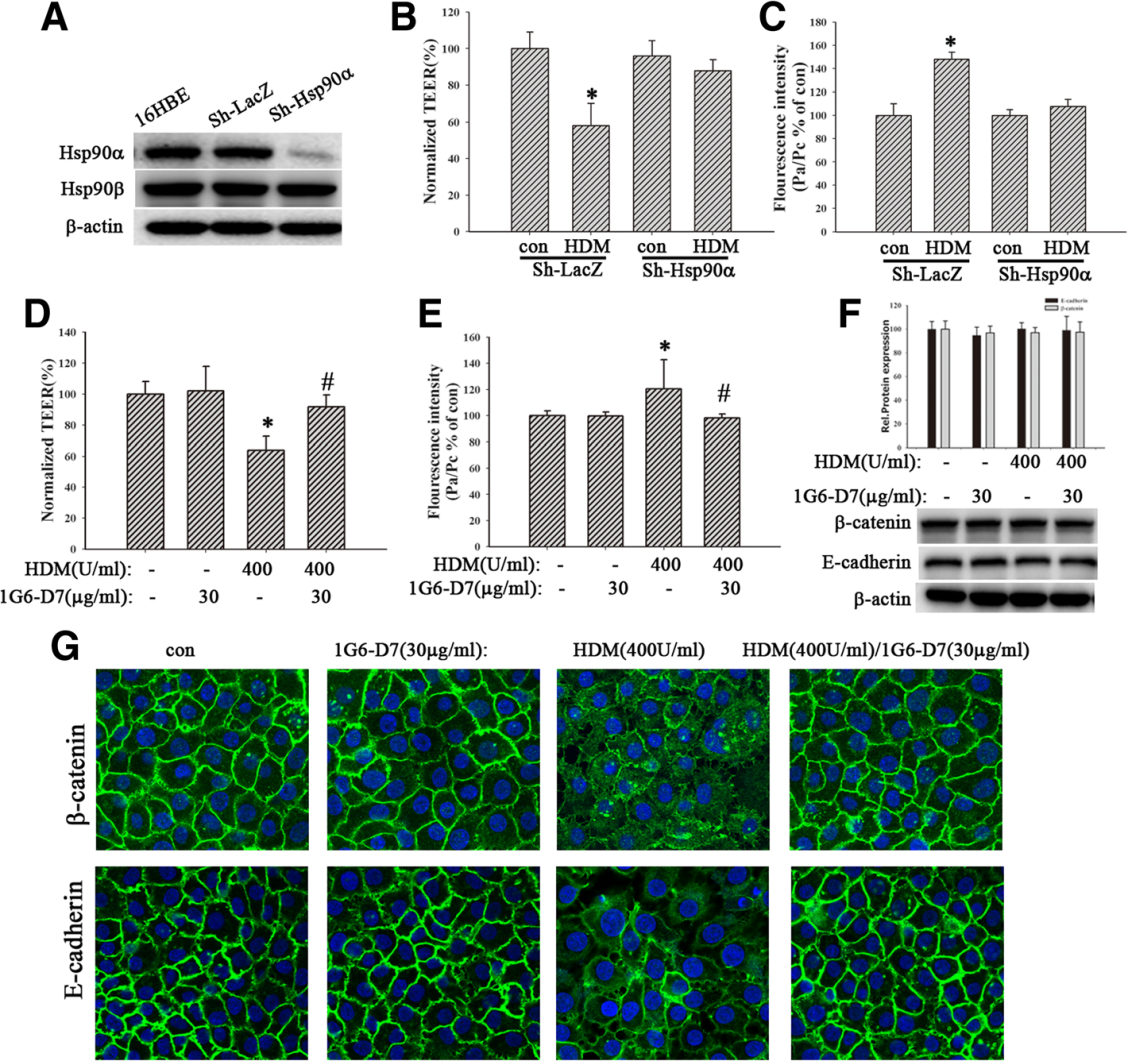
Both silencing of Hsp90α and the eHsp90α mAb, 1G6-D7, restored HDM-induced epithelial barrier dysfunction. **a** The expression of Hsp90α in sh-Hsp90α group decreased remarkably. **b** and **c** Sh-Hsp90α cells were treated with HDM (400 U/ml) for 24 h, then TEER values and FITC-DX were measured immediately. **d** and **e** Cells were pretreated with 1G6-D7 (30 μg/ml) for 2 h and then treated with HDM (400 U/ml) for 24 h. TEER values and FITC-DX were measured. **f**The expression of E-cadherin and β-catenin proteins was detected by western blotting analysis. **g** Immunofluorescence staining was used to show the localization of E-cadherin or β-catenin. Data are mean ± SD of four independent experiments.^*^ *P* < 0.05 versus con group; ^#^ *P* < 0.05 versus HDM group

Other studies showed that Hsp90 inhibitors protected and restored the loss of microvascular endothelial and intestinal epithelial barrier integrity [10, 44]. Our data above also demonstrated that Hsp90α played a key role in HDM-induced barrier dysfunction. To further evaluate the role of Hsp90α in HDM-induced barrier dysfunction, we successfully constructed lentivirus system for Hsp90α-silencing in 16HBE cells (Fig. 3a). The sh-Hsp90α cells were grown on transwell plates and then were exposed to HDM (400 U/ml) for 24 h. Results showed that Hsp90α-silencing alleviated HDM-induced barrier dysfunction by improving TEER values and FITC-DX (Fig. 3b-c, *P* < 0.05). Collectively, these results demonstrated that Hsp90α mediated HDM-induced bronchial epithelial barrier dysfunction.

### 1G6-D7restored HDM-induced bronchial epithelial barrier dysfunction

1G6-D7, a newly generated mAb which selectively targets the dual lysine region in secreted Hsp90α, recognizes and neutralizes the eHsp90α function [30, 31]. To identify that1G6-D7 inhibited HDM-induced airway epithelial barrier disruption via blocking eHsp90α function, cells were pretreated with1G6-D7 (30 μg/ml) for 2 h and following stimulated by HDM (400 U/ml) for 24 h. Results indicated that 1G6-D7 pretreatment exhibited a significant attenuation in the fall of TEER value (Fig. 3d, *P* < 0.05) and the raise of FITC-DX (Fig. 3e, *P* < 0.05), suggesting that eHsp90α was involved in the HDM-induced epithelial hyperpermeability. Furthermore, the immunofluorescence also revealed that 1G6-D7 restored HDM-induced delocalization of E-cadherin and β-catenin (Fig. 3g), instead of affecting their expression (Fig. 3f, *P* > 0.05).

### Inhibition of RhoA/MLC signaling alleviated bronchial epithelial barrier dysfunction

**Fig. 4:**
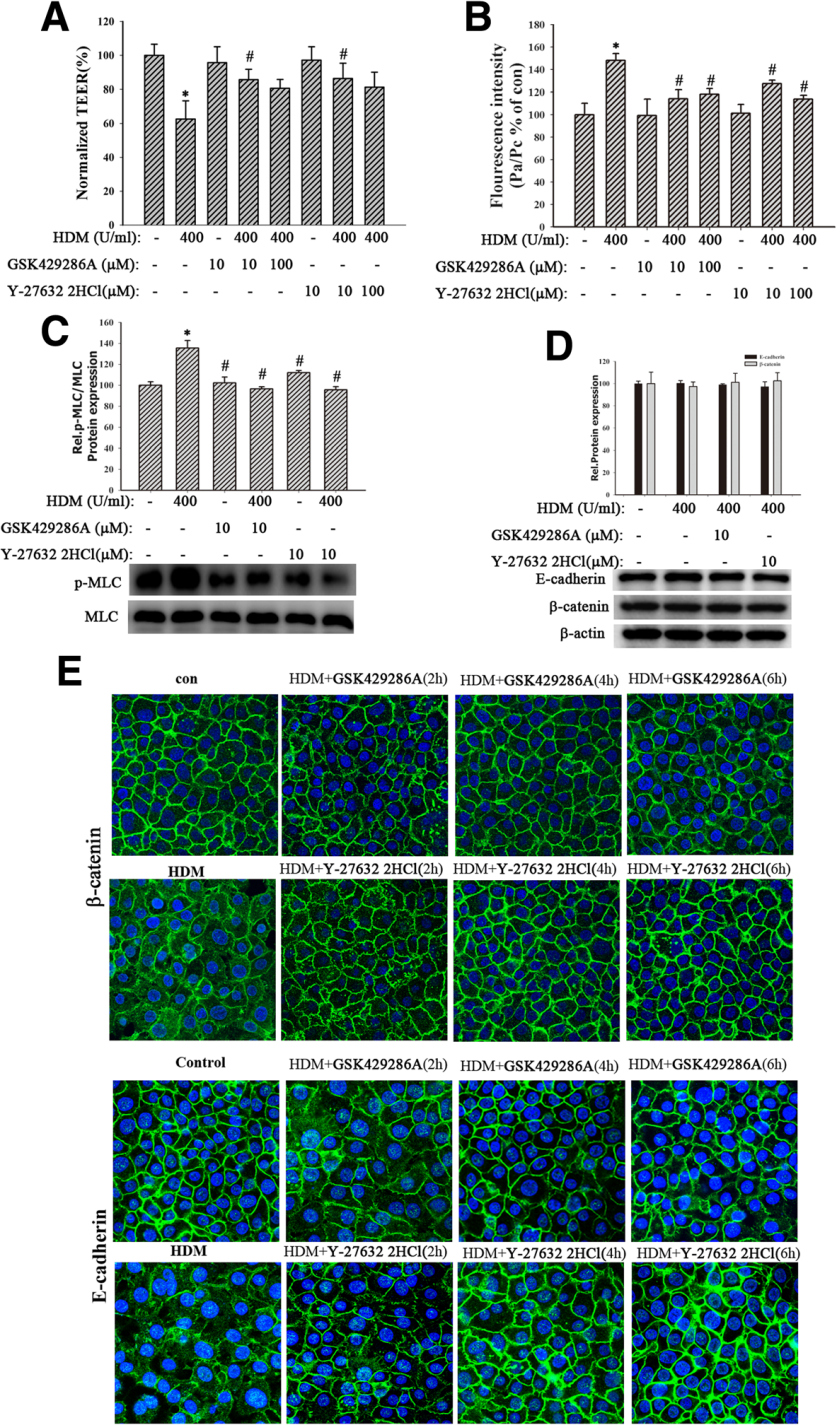
Inhibition of RhoA/MLC signaling alleviated HDM-induced bronchial epithelial barrier dysfunction. **a** and **b** Cells pretreated for 6 h with inhibitors of RhoA/MLC signaling, Y-27632 2HCl (10, 100 μM) and GSK429286A (10, 100 μM), were exposed to HDM (400 U/ml) for 24 h. TEER values and FITC-DX were measured immediately. **c** MLC and p-MLC were detected. **d** The expression of E-cadherin and β-catenin was detected. Data are mean ± SD of three independent experiments. **e** The location of E-cadherin and β-catenin was detected by immunofluorescence staining. Data are mean ± SD of four independent experiments.^*^ *P* < 0.05 versus con group; ^#^ *P* < 0.05 versus HDM group

RhoA and Rho kinase were involved in lysophosphatidic acid- and thrombin-induced endothelial barrier dysfunction [33, 34, 45]. Hsp90 inhibitors prevented LPS-induced endothelial barrier dysfunction by disrupting RhoA signaling [11]. Thus, we inferred that RhoA signaling might mediate the involvement of eHsp90α in HDM-induced bronchial epithelial barrier dysfunction. Cells were pretreated by the Rho kinase inhibitors, Y-27632 2HCl (10, 100 μM) and GSK429286A (10, 100 μM), for 6 h and then stimulated with HDM (400 U/ml) for 24 h. Results showed that both inhibitors restored the fall of TEER and the raise of FITC-DX (Fig. 4a-b, *P* < 0.05). Besides, cells were pretreated with Y-27632 2HCl (10 μM) or GSK429286A (10 μM) for 2, 4 or 6 h and then exposed to HDM (400 U/ml) for 24 h. The immunofluorescence revealed that HDM induced the discontinuity and diffusing of E-cadherin and β-catenin, which were restored by Rho kinase inhibitors (Fig. 4e). The protein expression of E-cadherin and β-catenin had no statistical difference in both Rho kinase inhibitors groups (Fig. 4d, *P* > 0.05). These data demonstrated RhoA/MLC signaling may mediate the involvement of eHsp90α in HDM-induced bronchial epithelial barrier dysfunction.

### Both HDM and hrHsp90α induced RhoA activity and MLC phosphorylation

**Fig. 5:**
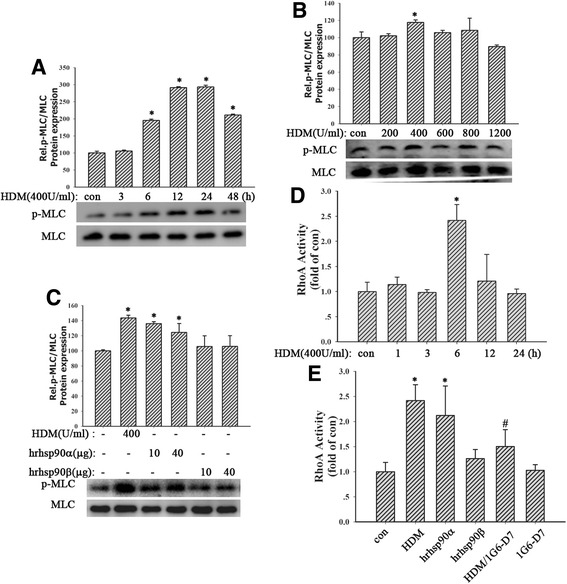
HDM and hrHsp90α increased the levels of RhoA activity and MLC phosphorylation. **a** and **b** 16HBE cells were treated with HDM (200, 400, 600, 800, or 1200 U/ml) for 24 h or stimulated with HDM (400 U/ml) for 3, 6, 12, 24, or 48 h. MLC and p-MLC were detected. **c** Cells were exposed to HDM (400 U/ml), hrHsp90α (10, 40 μg/ml) or hrHsp90β (10, 40 μg/ml) for 24 h. MLC and p-MLC were detected. **d** Cells exposed to HDM (400 U/ml) for 1, 3, 6, 12, 24 h. Then RhoA activity was measured by Rho G-LISA RhoA activation assay kit^TM^ biochem kit. **e** 16HBE cells pretreated with 1G6-D7 (30 μg/ml) for 2 h and then stimulated with HDM (400 U/ml), hrHsp90α (10 μg/ml) or hrHsp90β (10 μg/ml) for 6 h. RhoA activity was measured. Data are mean ± SD of three independent experiments.^*^ *P* < 0.05 versus con group; ^#^ *P* < 0.05 versus HDM group

To clarify the involvement of RhoA/MLC signaling in loss of bronchial epithelial barrier integrity, cells were exposed to different concentrations of HDM (200,400, 600, 800 or 1200 U/ml) for 24 h or HDM (400 U/ml) for indicated time (3, 6,12, 24 or 48 h). Western blotting analysis indicated that HDM (400 U/ml) increased MLC phosphorylation beginning 6 h posttreatment, which remained elevated for many hours (Fig. 5a-b, *P* < 0.05). Meanwhile, cells were pretreated by Y-27632 2HCl (10 μM) and GSK429286A (10 μM) respectively for 6 h before treatment with HDM (400 U/ml) for 24 h. Results showed that both inhibitors decreased the expression of p-MLC (Fig. 4c, *P* < 0.05). Similarly, 16HBE cells exposed to hrHsp90α (10, 40 μg/ml) or hrHsp90β (10, 40 μg/ml) for 24 h. The results revealed that hrHsp90α instead of hrHsp90β induced MLC phosphorylation (Fig. 5c, *P* < 0.05). Furthermore, we also found that HDM (400 U/ml) increased RhoA activity at 6 h. Similarly, hrHsp90α (10 μg/ml) instead of hrHsp90β (10 μg/ml) also promoted the activation of RhoA signaling (Fig. 5d-e, *P* < 0.05). These data demonstrated that HDM and hrHsp90α activated RhoA/MLC signaling, contributing to bronchial epithelial barrier dysfunction.

### Inhibition of Hsp90α suppressed RhoA activity and MLC phosphorylation

**Fig. 6:**
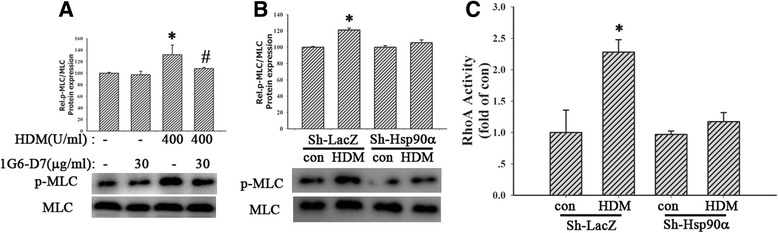
Inhibition of Hsp90α suppressed RhoA activity and MLC phosphorylation. **a** Pretreated with 1G6-D7 (30 μg/ml) for 2 h, cells were exposed to HDM (400 U/ml) for 24 h. MLC and p-MLC were detected. **b** and **c** Sh-Hsp90α cells were exposed to HDM (400 U/ml) for 24 h. the activity of RhoA and the phosphorylation of MLC were measured respectively. Data are mean ± SD of three independent experiments.^*^ *P* < 0.05 versus con group; ^#^ *P* < 0.05 versus HDM group

In order to further investigate the exact relationship between eHsp90α and RhoA/MLC signaling, cells were pretreated by 1G6-D7 (30 μg/ml) for 2 h, then stimulated by HDM(400 U/ml), hrHsp90α (10 μg/ml) or hrHsp90β (10 μg/ml) for 24 h. Results showed that 1G6-D7 attenuated HDM-induced RhoA activity (Fig. 5e, *P* < 0.05). Next we found that HDM induced robust MLC phosphorylation at 24 h, while it was partly blocked by 1G6-D7 (30 μg/ml) (Fig. 6a, *P* < 0.05), suggesting that inhibition of eHsp90α suppressed HDM-mediated MLC phosphorylation. Similarly, Hsp90α-silencing suppressed HDM-induced RhoA activity (Fig. 6c, *P* > 0.05) and MLC phosphorylation (Fig. 6b, *P* > 0.05). Collectively, these data demonstrated that RhoA/MLC signaling was the downstream of eHsp90α.

## Discussion

In this study, we first propose a novel mechanism that eHsp90α mediates HDM-induced human bronchial epithelial barrier dysfunction via activating RhoA/MLC signaling.

Epithelial hyperpermeability and disruption of AJ proteins are used for assessing the epithelial barrier dysfunction. Hyperpermeability of bronchial epithelium can result in greater penetration of inhaled allergens and particles into the subepithelial space, facilitating antigen sampling and innate and adaptive immune responses [46]. AJs are important for initiation and maintenance of cell-cell adhesion and are involved in numerous signal transduction cascades [47, 48]. We previously identified that mice sensitized and challenged with 400 U/ml HDM could exhibit the typical features of atopic asthma: AHR, high serum IgE and eosinophilic airway inflammation [41]. In clinic, HDM(100–100,000 U/ml, Alutard) is mainly used for immunotherapy [49]. In this study, the concentrations of HDM we used ranged from 200 U/ml to 1200 U/ml, which also complied with the clinical application. Here we found that HDM increased epithelial permeability and disrupted the integration of E-cadherin/β-catenin complex in the membrane, but didn’t affect their expression. The original TEER value in HDM(400 U/ml) group (15.87 ± 1.7 Ω.cm^2^) decreased compared to con group (25.2 ± 1.1 Ω.cm^2^), and the original lower chamber fluorescence value of FITC-DX in HDM(400 U/ml) group (425.50 ± 13.92 a.u.) increased compared to con group (358.67 ± 12.01 a.u.). These results were in agreement with the study that HDM did not affect expression of E-cadherin protein [50] but disagreement with another study that HDM induced the downregulation of E-cadherin expression [40]. Consistently, we also identified that HDM promoted the distribution of E-cadherin and β-catenin. It indicated that the locations of E-cadherin and β-catenin were more essential to maintain cell-cell adhesion than the level of proteins expression. Collectively, our results revealed that HDM induced the delocalization of E-cadherin and β-catenin, promoting the defect of bronchial epithelial barrier function.

For the exact mechanisms of bronchial epithelial barrier dysfunction induced by HDM are still largely unknown, we first demonstrated the involvement of eHsp90α in this process. Hsp90 plays a critical role in many biological processes, such as inflammation and barrier function [14–16]. Early studies also indicated that Hsp90 mRNA and protein expression were increased in asthmatic patients [13, 14], suggesting the potential role of Hsp90 in asthma. In this study, we observed that HDM increased the secretion of Hsp90α in 16HBE cells, coupled with the upregulation of Hsp90α protein. Catravaset al demonstrated that Hsp90 mediated endothelial barrier dysfunction [11, 44]. Inhibition of Hsp90 improved the loss of microvascular endothelial and intestinal epithelial barrier integrity [10, 44], indicating the involvement of Hsp90 in barrier disruption. To our knowledge, Hsp90β mainly acts as a molecular chaperone protein and is essential for life [17, 18]. While Hsp90α secreted by cells exerts pro-mobility signal, which is involved in wound healing and tumor invasion [9]. Therefore, our study focused on the effect of Hsp90α on bronchial barrier function. This study found that defects in barrier structure and function were observed in human bronchial epithelial monolayers stimulated with hrHsp90α instead of hrHsp90β, paralleled with the increasing expression and secretion of Hsp90α in HDM-treated 16HBE cells. These results indicated that Hsp90α was critical in HDM-induced bronchial barrier dysfunction. To further explore the exact role of Hsp90α on airway barrier dysfunction induced by HDM, we successfully knocked down the protein expression of Hsp90α in 16HBE cells by using lentivirus system technique. Expectedly, Hsp90α-silencing cells exhibited a remarkable improvement of epithelial hyperpermeability induced by HDM, suggesting that HDM caused barrier dysfunction partly in an Hsp90α-dependent manner. Besides, many studies have showed that eHsp90 participates in TGF-β-mediated collagen production in myocardial fibroblasts [23], wound healing in keratinocytes [9, 24] and contributes to tumor growth, invasion, and inflammatory storm [9, 25–29]. Taken together, these prompted us to infer that eHsp90α may play a critical role on HDM-induced bronchial barrier defects. 1G6-D7, a specific anti-secreted Hsp90α mAb, recognizes and neutralizes eHsp90α function [30, 31]. In this study, 1G6-D7 pretreatment restored the fall of TEER and the raise of FITC-DX and the delocalization of E-cadherin and β-catenin, suggesting that eHsp90α mediated bronchial epithelial barrier dysfunction induced by HDM. However, intracellular Hsp90α may also be involved in the dysfunction of bronchial epithelial barrier, which needs to be clarified by specifically blocking the intracellular Hsp90α without affecting eHsp90α.

We have identified that eHsp90α mediates HDM-induced barrier dysfunction. However, the mechanism that eHsp90α induce the disruption of the epithelial barrier is not well defined. Catravas JD, et al. reported Hsp90 inhibitors prevented LPS-induced endothelial barrier dysfunction by disrupting RhoA signaling [11], indicating that the RhoA/Rho-kinase pathway activated by Hsp90 could result in the endothelial barrier disruption. RhoA signaling plays important roles in many cellular functions, including contraction, motility and proliferation and barrier dysfunction [32–34]. Inhibition of Rho kinase reduced MLC phosphorylation, a target of the Rho/ROCK pathway, thus producing airway relaxation [51]. Cigarette smoke caused AJC disruption and increased epithelial permeability via triggering the RhoA/ROCK signaling pathway in bronchial epithelial cells [39]. In agreement with these findings, our study also found both HDM increased RhoA activity at 6 h and promoted MLC phosphorylation beginning 6 h, which remained elevated for many hours. Both Rho kinase inhibitors, Y-27632 2HCl and GSK429286A, restored epithelial hyperpermeability and AJC disassembly induced by HDM and hrHsp90α. Consistently, hrHsp90α instead of hrHsp90β increased RhoA activity and MLC phosphorylation. We further observed that inhibition of eHsp90α by1G6-D7 or Hsp90α-silencing suppressed the activation of RhoA/MLC signaling induced by HDM, suggesting that eHsp90α secreted by HDM-treated cells increased epithelial permeability and promoted AJC disruption through RhoA/MLC signaling activation, leading to a loss of epithelial cell-cell contact. Previous study reported that RhoA signaling promoted endothelial MLC phosphorylation, cytoskeletal reorganization and thereby increased endothelial stress fiber formation, gap formation, and protein permeability [36]. Thus it may affect the organization of cytoskeleton anchoring with the plasma membrane and AJCs, contributing to the barrier dysfunction.

Here, we found that HDM induced RhoA activity at 6 h and MLC phosphorylation on Ser19/Thr18 began at 6 h and persisted for at least 18 h in bronchial epithelial cells. These results disagreed with the results observed in endothelial cells exposed to thrombin [33, 34, 52] or LPS [11]. The thrombin-induced MLC phosphorylation was acute (began at 5–10 min) and transient (persisted for 1 h), which was correlated with the transiently inactivated MLC phosphatase and a peak in Ca^2+^ mobilization to produce maximal MLC phosphorylation [52]. On the other hand, LPS-induced RhoA activity at 2 h and MLC phosphorylation began at 2 h and persisted for hours partially in a Hsp90-dependent manner [11]. However, the results showed HDM induced RhoA activation and MLC phosphorylation partly through eHsp90α secretion. Yet other mechanisms involved should also be considered. The receptor LRP-1, connecting eHsp90α signaling to serine 473 phosphorylation in Akt kinase [53], may contribute to the activation of RhoA/MLC signaling.

One phenomenon we have to explain is that HDM induced activation of RhoA/MLC signaling at 6 h while eHsp90α was detected at 24 h. On the one hand, the level of eHsp90α at earlier time (<24 h) is too little to detected by the method of concentration and purification of condition media. Thus we can’t compare the level of eHsp90α between con and HDM groups. On the other hand, we detected that the Hsp90α protein expression in HDM-treated cells remarkably increased at 6 h. Most importantly, both blocking eHsp90α by 1G6-D7 and knocking down Hsp90α protein expression inhibited the activity of RhoA and the phosphorylation of MLC induced by HDM, indicating that eHsp90α mediated the activation of RhoA/MLC signaling.

However, one limitation of this study is the use of 16HBE cells rather than primary bronchial epithelial cells from donors (health or asthma), which are also important for providing more evidences to validate our findings.

## Conclusion

In summary, we first demonstrate that HDM increases the production of eHsp90α and activates RhoA/MLC signaling in16HBE cells. Neutralization of eHsp90α by 1G6-D7inhibits the activation of RhoA/MLC and ameliorates bronchial epithelial barrier dysfunction induced by HDM. Moreover, inhibition of RhoA/MLC signaling prevents and restores epithelial hyperpermeability and AJC disruption induced by HDM or hrHsp90α, indicating the critical role of RhoA/MLC signaling in bronchial barrier dysfunction. Collectively, eHsp90α mediates HDM-induced human bronchial epithelial barrier dysfunction partially by activating RhoA/MLC signaling. And the newly generated mAb, 1G6-D7, which selectively targets the dual lysine region in secreted Hsp90α, could be a novel agent for the treatment of asthma.

## Abbreviations

16HBE: Human bronchial epithelial cell line 16HBE14o-
AJC: AJs complex
AJs: Adherens junctions
ANOVA: One-way analysis of variance
DAPI: 4′,6-diamidino-2-phenylindole dihydrochloride
EHsp: 90α extracellularhsp90α
FITC-DX: FITC-dextran flux
HDM: House dust mite
Hr: Human recombinant
HRP: Horseradish peroxidase
Hsp: Heat shock protein
MAb: Monoclonal antibody
MLC: Myosin light chain
p-MLC: Phospho-MLC
ROCK: Rho kinase
SD: Means ± standard deviations
TEER: Transepithelial electrical resistance
TJs: Tight junctions
